# The Mechanism of Adsorption of Rh(III) Bromide Complex Ions on Activated Carbon

**DOI:** 10.3390/molecules26133862

**Published:** 2021-06-24

**Authors:** Marek Wojnicki, Andrzej Krawontka, Konrad Wojtaszek, Katarzyna Skibińska, Edit Csapó, Zbigniew Pędzich, Agnieszka Podborska, Przemysław Kwolek

**Affiliations:** 1 Faculty of Non-Ferrous Metals, AGH University of Science and Technology, Mickiewicza Ave. 30, 30-059 Krakow, Poland; krawontk@student.agh.edu.pl (A.K.); wojtaszek@student.agh.edu.pl (K.W.); kskib@agh.edu.pl (K.S.); 2 MTA-SZTE Biomimetic Systems Research Group, University of Szeged, H-6720 Dóm tér 8, 6720 Szeged, Hungary; tidecs2000@yahoo.co.uk; 3 Interdisciplinary Excellence Centre, Department of Physical Chemistry and Materials Science, University of Szeged, Rerrich B. tér 1, H-6720 Szeged, Hungary; 4 Faculty of Materials Science and Ceramics, AGH University of Science and Technology, al. A. Mickiewicza 30, 30-059 Krakow, Poland; pedzich@agh.edu.pl; 5 Academic Centre for Materials and Nanotechnology, AGH University of Science and Technology, al. A. Mickiewicza 30, 30-059 Krakow, Poland; podborsk@agh.edu.pl; 6 Department of Materials Science, Faculty of Mechanical Engineering and Aeronautics, Rzeszow University of Technology, 35-959 Rzeszow, Poland; pkwolek@prz.edu.pl

**Keywords:** activated carbon, rhodium adsorption, recycling, recovery, sorption

## Abstract

In the paper, the mechanism of the process of the Rh(III) ions adsorption on activated carbon ORGANOSORB 10—AA was investigated. It was shown, that the process is reversible, i.e., stripping of Rh(III) ions from activated carbon to the solution is also possible. This opens the possibility of industrial recovery of Rh (III) ions from highly dilute aqueous solutions. The activation energies for the forward and backward reaction were determined These are equal to c.a. 7 and 0 kJ/mol. respectively. Unfortunately, the efficiency of this process was low. Obtained maximum load of Rh(III) was equal to 1.13 mg per 1 g of activated carbon.

## 1. Introduction

Rhodium, Rh, is a silvery-white noble metal belonging to the platinum group metals, PGM. It is highly corrosion-resistant, and, similarly to iridium and contrary to platinum, palladium and osmium does not react with *aqua regia*. Rhodium is an extremely rare metal. Its concentration in the earth’s crust equals 10^−1^ ppb. Usually, it is found together with other PGMs. Metallic rhodium is a by-product of the extraction of nickel and copper from their ores [1]. The vast majority of the world’s rhodium resources is probably located in Bushveld Complex in the Republic of South Africa, RSA. This country is also the main supplier of this metal. In 2019 it provided 18 tons of Rh which accounts for *c.a.* 90% of the world production [2]. In 2020 lower quantity of rhodium was produced due to some technical problems of the biggest Rh-supplier, Anglo American Platinum (Amplats), and a very strict lockdown implemented to prevent COVID-19 transmission among miners in RSA [3]. This affected the price of the metal (*vide infra*). Other Rh deposits are located in Canada, the USA, Colombia, and Russian Federation [4].

**Figure 1:**
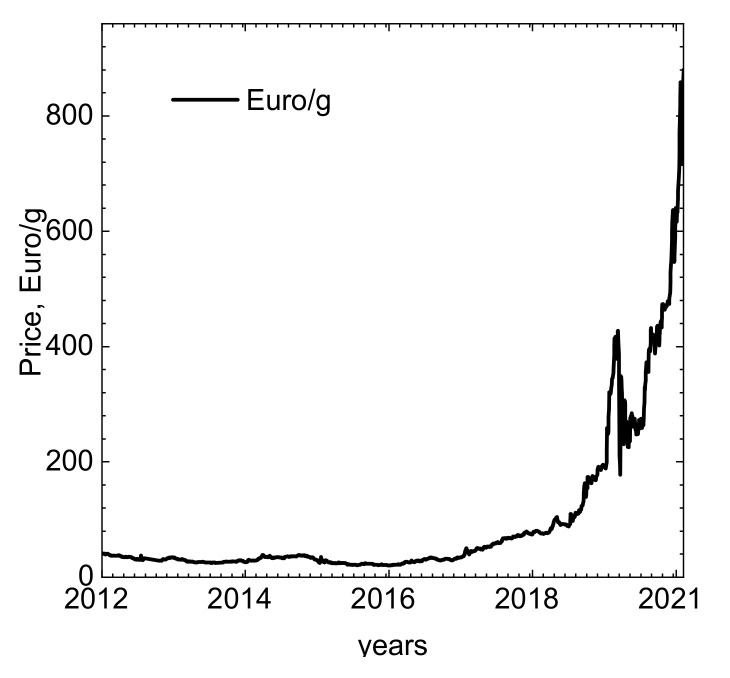
Rhodium price evolution over the last decade [10].

Rhodium is added to platinum to improve its hardness and reduce the thermal expansion coefficient. Pt-Rh alloys are used for fabrication of crucibles for laboratory furnaces, spark plug electrodes, thermocouples, also in jewelry, glass industry, electronics, and electroplating. [1]. The most important, however, is fabrication of catalysts working at the elevated temperature such as catalytic converters for the automotive industry. This consumes around 80% of rhodium supplies. [5,6]. Due to more and more strict environmental regulations regarding NO*_x_* emission and high demand for gasoline vehicles in developing countries, such as the People Republic of China, consumption of rhodium increases gradually, but the supplies not. Thus, the price of Rh increased rapidly during last year (Figure 1). Consequently, this stimulates the development of recycling processes. Recycling has already become an important source of rhodium supplies. As long ago as in 2010, rhodium recycling accounted for 7 tons of metal [7]. Even further increase of rhodium demand in the future can be speculated knowing that its organic complexes are promising light-emitting material in OLEDs [8,9].

Rhodium, due to its high corrosion resistance, can be dissolved in acidic solution only when it is in the form of rhodium(III) oxide, Rh_2_O_3_. Thus, the first step of the recycling process is roasting the host material of the catalytic converter in the air. Then rhodium oxide isdissolved, recovered and refined to obtain pure metal [7]. The recovery can be achieved using various methods e.g., precipitation with polyamines [11], solvent extraction, extraction with resins [12,13,14] and zinc cementation [15]. Very often, the recovery process determines the economic efficiency of the recycling of PGMs. Thus, it deserves appropriate scientific attention to study and develop the recovery methods. Besides, environmental factors must also be taken into account. The most important recovery methods, such as solvent extraction [16], ion exchange [12], cementation [17], electrowinning [18] and adsorption [19] are well known since years. Their applicability, however, strongly depends on the chemical composition of the leachate [13,20]. Thus, they should be adapted to the specific conditions of the recycling process.

The literature on the problem of Rh extraction from aqueous solutions is scarce, possibly due to low rhodium prices over the last decades. Solvent extraction of Rh is possible using organic extractors [14,21,22]. This method, however, is still too expensive for widespread industrial use. Ion exchange, in turn, with coal or resin [23] as an absorbent is time-consuming [24]. Cementation, relatively cheap and rapid, is readily used in industry. This is simply a reduction process, with a metal less noble as rhodium as the reducing agent This is usually applied in the form of a fine powder. Unfortunately, the reducing agent becomes the contaminant of the extracted metal. Thus, Rh of higher purity is obtained when the reduction is performed in a homogeneous system, using formic acid, hydrazine hydrate, hydrazine sulfate [15], or ferrous ions as the reducing agent [25]. Electrowinning, in turn, is not suitable for highly diluted solutions [26]. Interestingly, the adsorption of rhodium ions was rarely studied. Rhodium chloride complex ions were adsorbed onto activated carbon [27] andTiO_2_. under AM1 simulated sunlight illumination [28].

In this work, we decided to explore the possibility of adsorption of Rh(III) bromide complex ions on activated carbon. This method was extensively studied for the recovery of noble metals, mainly gold [29,30] and platinum [31,32,33]. Bromide anions were selected as the complexing agent because the stability of the complex ion is lower when compared to the chloride one [34,35]. This offers, at least theoretically, the possibility of adsorption and subsequent reduction of Rh(III) to the metallic form as it was observed in the case of Au(III) chloride complex ions [36,37,38,39]. Such a process is irreversible and offers a higher recovery yield when compared to the physical adsorption, where a certain equilibrium is established between the species adsorbed and remaining in the solution.

The TD-DFT calculation method was used to calculate theoretical UV-Vis spectra of adsorbed species. The TD-DFT methods are often applied for IR, Raman, UV-Vis spectra calculations as well as for molecule structure optimization [40,41,42,43]. In the case of systematic studies, it may also help to understand the mechanism of the reaction.

This is also the first step towards the systematic studies of adsorption of various complexes of noble metals onto activated carbon. The aim of such a study is a validation of a recently introduced adsorption isotherm which seems to be useful for modeling the industrially important processes of noble metals recovery from leachate.

## 2. Experimental

Rh(III) bromide complex ions were prepared according to the following procedure. Firstly, 2.0152 g of metallic rhodium (Onyx Met, 99.97 wt.%) was placed in a round bottom flask and 40 mL of the concentrated, 95 wt.%, sulfuric(VI) acid of laboratory grade, together with 20 mL of deionized (DI) water (>18 MΩ, Polwater), were slowly added. The reactor was heated up to 340°C in the sand bath, after c.a. 5 days the metal was dissolved and the brown solution was obtained. This was cooled to room temperature and diluted to 2 L with DI water. The color of the solution changed to yellow-brown.

Secondly, sodium carbonate (Eurochem, laboratory-grade) was added dropwise to the Rh-containing solution to obtain rhodium(III) carbonate. Although no immediate precipitation was observed, the electrical conductivity of the solution was decreasing gradually. The process was stopped when the electrical conductivity started increasing. After continuous stirring for 48 h at room temperature, the precipitate, Rh_2_(CO_3_)_3_, was formed. Then it was filtered, washed with DI water, dried and weighted. The overall yield of this process, was approximately 72.5%.

Finally, 0.384 g of rhodium(III) carbonate was placed in a volumetric flask made of borosilicate glass, partially filled with 0.1 M hydrobromic acid. Then the reaction mixture was heated up to 95 °C and stirred using a quartz rod for 210 min. A transparent solution containing Rh(III) bromide complex ions was obtained.

Rh(III) bromide complex ions were adsorbed on activated carbon from 0.1 M HBr solutions, pH = 1, The volume of the solution applied in the experiments was 200 mL. Temperature T was between 40 and 70 °C and was controlled using a water bath. The solution was agitated using a magnetic stirrer, the stirring rate was usually 1200 rpm. Rh initial concentration in the adsorption experiments, [Rh(III)]_0_, was between 1 × 10^−4^ M and 8 × 10^−4^ M. Its changes were monitored using Shimadzu 2501PC spectrophotometer. This was also verified using microwave plasma atomic emission spectrometer MP-AES (Agilent 4200). Standard solution with Rh concentration of 1000 ± 7 µg/mL was obtained from SCP Science Solution and was appropriately diluted. The amount of activated carbon used for adsorption tests [C] was between 0.2 and 1 g. A desorption test was conducted in 200 mL of 0.1 M HBr. Rh(III) bromide complex ions concentration in the solution was measured spectrophotometrically.

Activated carbon, ORGANOSORB 10—AA (Desotec, Poland) was used as an adsorbent. Its specific surface area was 917 m^2^/g. This was determined with the Brunauer–Emmett–Teller (BET) method (Quantachrome, model Nova 1200). Nitrogen gas was used as the adsorbate. It was assumed that a single nitrogen molecule can cover 0.16 nm^2^ of the substrate area. Prior adsorption test, sample, 0.5331 g, was degassed for 24 h at 120 °C. At this temperature also the water was removed.

The specimen of activated carbon with adsorbed rhodium species for further analysis was obtained with the following experimental conditions: stirring rate 1200 rpm, [C] = 0.5 g, [Rh(III)]_0_~1.1 × 10^−4^ M, pH = 1, [Br]^−^ = 0.1 M T = 50 °C. Its chemical composition was determined using Scanning Electron Microscope (SEM) SEM JEOL—6000 Plus equipped with Energy-Dispersive X-Ray Spectroscopy (EDS) analyzer (EX-37001). The samples were glued on a carbon disc. One of the samples was mechanically polished using the emery paper with grit number P1200 prior microscopic examination to remove its outer layer.

Further characterization of the adsorbent was done using X-ray photoelectron spectroscopy, XPS in three areas of the specimen. XP spectra were recorded on a PHI 5000 VersaProbe II (ULVAC-PHI, Chigasaki, Japan) system using a microfocused (100 μm, 25 W) Al Kα X-ray beam with a photoelectron take-off angle of 45°. A dual-beam charge neutralizer was used to compensate for the charging effect. The operating pressure in the analytical chamber was less than 2 × 10^−9^ mbar. High-resolution spectra were collected with an analyzer pass energy of 11.75 eV. All XPS peaks were referenced to the neutral (C-C) carbon C1s peak at 284.8 eV. Spectrum background was subtracted using the Shirley method.

The TD-DFT calculation method was used to calculate theoretical UV-Vis spectra of Rh(III) species to compare to the experimental ones. The TD-DFT methods are often applied for molecule structure optimization [40,41,42,43]. Geometrical optimizations were performed using the B3LYP (the Becke three-parameter-Lee–Yang–Parr) functional and SDD base. Single point energies using solvent corrections at the CPCM level with water as a solvent was used. Molecular orbitals and surfaces were plotted from GausView5 software [44]. UV-Vis spectra were deconvoluted using Origin b9.55.409 software.

## 3. Results and Discussion

### 3.1. Development of the Model Describing the Reaction between Rh(III) Bromide Complex Ions with Activated Carbon

**Figure 2:**
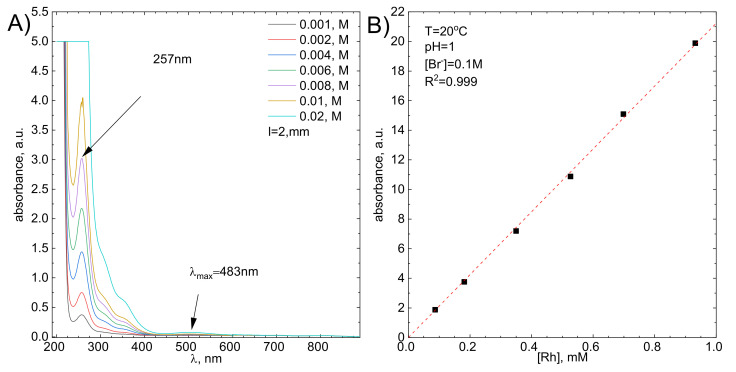
(**A**) UV-Vis spectra of the studied solution as a function of Rh initial concentration, (**B**) Molar absorption coefficient determination using graphical method.

The kinetics of Rh(III) bromide complex ions adsorption/reduction was studied spectrophotometrically, at wavelength λ = 257 nm, because this is the strongest absorption band in the spectrum (Figure 2A). Its molar absorption coefficient, ε = 21,229.99 ± 162.46$\left[\frac{dm^{3}}{mol\cdot cm}\right]$, was determined experimentally using the Lambert-Beer law (Figure 2B).

**Figure 3:**
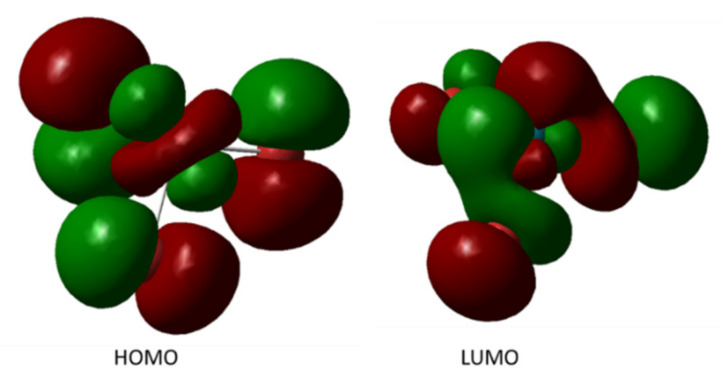
The HOMO and LUMO orbital for RhBr_3_ complex.

Analysis of the results of TD-DFT calculations indicated that the recorded spectra correspond to RhBr_3_ complex ions (see Supplementary Information Figure S1 and Table S1). The energy of their HOMO and LUMO orbitals was also calculated (Figure 3).

**Figure 4:**
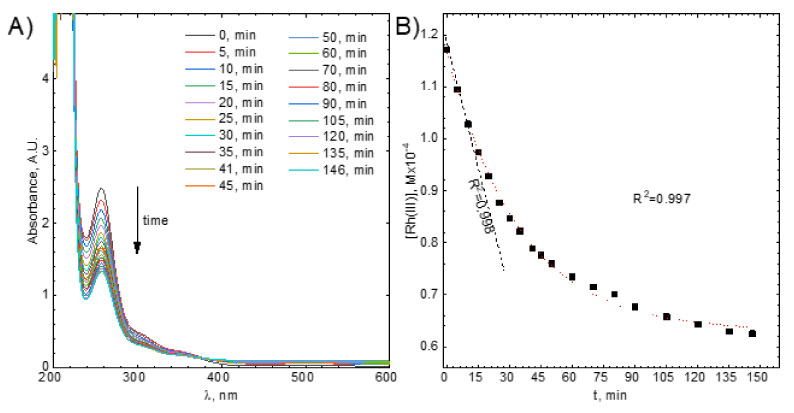
(**A**) UV-Vis spectra evolution *vs.* time, (**B**) example of the kinetic curve with the fitted integral form of the kinetic equation (dotted line) and straight line, the dashed one, showing the initial reaction rate. Experimental conditions: [C] = 1 g, [Rh(III)] ≈ 1.1 × 10^−4^ M, T = 50 °C, V = 1200 rpm.

Next, the absorbance was converted to Rh(III) bromide complex ions concentration, [Rh(III)], and the kinetic curve was plotted (Figure 4B). It has a specific exponential shape and tends asymptotically to a certain value. The latter suggests the reversibility of the studied process. In other words, an equilibrium state is probably achieved. This can be described using Equation (1):

(1) $${\left[Rh(III)\right]}_{}+[C]\overset{k_{f}}{\underset{k_{b}}{⇌}}{\left[Rh(III)\right]}_{ads}$$

where [Rh(III)] and [Rh(III)]_ads_ describe concentrations of Rh(III) bromide complex ions in the solution and adsorbed on the surface of activated carbon respectively, and k_f_ and k_b_ are forward and backward reaction rate constants. The reaction order was assumed as equal to 1. The change in carbon concentration was neglected. Under these conditions, the following equation is also valid (Equation (2)):

(2) $${\left[Rh(III)\right]}_{0}+{\left[C\right]}_{0}+{\left[Rh(III)\right]}_{ads,0}={\left[Rh(III)\right]}_{t}+{\left[C\right]}_{t}+{\left[Rh(III)\right]}_{ads,t}={\left[Rh(III)\right]}_{eq}+{\left[C\right]}_{eq}+{\left[Rh(III)\right]}_{ads,eq}$$

where subscript “t” corresponds to any time during the experiment, “eq” to the equilibrium state and “0” to the initial concentration. Assuming isolation conditions, the reaction can be treated as a pseudo-first order (Equation (3)):

(3) $${\left[C\right]}_{0}\approx {\left[C\right]}_{t}\approx {\left[C\right]}_{eq}$$

Also, the equilibrium constant can be given in the following form, Equation (4):

(4) $$K=\frac{k_{f}}{k_{b}}=\frac{{\left[Rh(III)\right]}_{ads,eq}}{{\left[C\right]}_{eq}\cdot {\left[Rh(III)\right]}_{eq}}$$

The reaction rate can be described using the following differential equation (Equation (5)):

(5) $$-\frac{d{\left[Rh(III)\right]}_{t}}{dt}=k_{f}{\left[Rh(III)\right]}_{t}\cdot {\left[C\right]}_{t}-k_{b}\cdot {\left[Rh(III)\right]}_{ads,t}$$

Using Equations (2)–(4), Equation (5) can be rearranged to the following form, Equation (6):

(6) $$-\frac{d{\left[Rh(III)\right]}_{t}}{dt}=\left({\left[Rh(III)\right]}_{t}-{\left[Rh(III)\right]}_{eq}\right)\left(k_{f}\cdot {\left[C\right]}_{t}+k_{b}\right)$$

The observed rate constant of the process, R_0_, is defined as Equation (7):

(7) $$R_{0}=k_{f}\cdot {\left[C\right]}_{t}+k_{b}$$

Because activated carbon is not homogeneous material i.e., there are various functional groups on its surface, with different adsorption abilities, it is more reasonable to define observed rate constants for forward and backward reactions separately, Equations (8) and (9):

(8) $$k_{f,obs}\sim k_{f}\cdot {\left[C\right]}_{t}$$

and

(9) $$k_{b,obs}\sim k_{b}$$

Differential Equation (6), can be solved by a simple integration from t = 0 to t = t and from [Rh(III)]_t_ = [Rh(III)]_0_ to [Rh(III)]_t_ = [Rh(III)]_t_, Equation (10):

(10) $$\int_{{\left[Rh(III)\right]}_{t}={\left[Rh(III)\right]}_{0}}^{{\left[Rh(III)\right]}_{t}={\left[Rh(III)\right]}_{t}}\frac{d{\left[Rh(III)\right]}_{t}}{\left({\left[Rh(III)\right]}_{t}-{\left[Rh(III)\right]}_{eq}\right)}=-R_{0}\int_{t=0}^{t=t}dt$$

The integral form of Equation (10) is the following, Equation (11):

(11) $${\left[Rh(III)\right]}_{t}={\left[Rh(III)\right]}_{eq}+\left({\left[Rh(III)\right]}_{0}-{\left[Rh(III)\right]}_{eq}\right)\cdot e^{-R_{0}\cdot t}$$

The kinetic curves were fitted using this equation (see Figure 4B). [Rh(III)]_eq_ and R_0_ were determined directly from the fit whereas [Rh(III)]_0_ was known. When it was equal to 1.17 × 10^−4^ M the equilibrium concentration of Rh(III) bromide complex ions in the solution was 4.54 × 10^−5^ M. Because fitting gives only the R_0_ which is the sum of the observed rate constants for forward and backward reactions, the rate constants were determined using the initial rate method.

The rate of the reaction studied, Equation (1), is defined with Equation (12)

(12) $$V=\frac{d{\left[Rh(III)\right]}_{t}}{dt}=-k_{f,obs}{\left[Rh(III)\right]}_{t}+k_{b,obs}\cdot {\left[Rh(III)\right]}_{ads,t}$$

When t→0 the reaction rate becomes the initial rate V_0_; ${\left[Rh(III)\right]}_{t}\approx {\left[Rh(III)\right]}_{0}$${\left[Rh(III)\right]}_{ads,0}\approx 0$ and the initial rate of the process is given with Equation (13).

(13) $$V_{0}=\frac{d{\left[Rh(III)\right]}_{t}}{dt}=-k_{f,obs}{\left[Rh(III)\right]}_{0}$$

Thus, the initial part of the kinetic curve, i.e., the first three data points, were approximated using the straight line. Its slope is equal to the initial rate of the studied process, V_0_ (Figure 4B).

When ${\left[Rh(III)\right]}_{0}=1.17\cdot 10^{-4}M$, $V_{0}=1.42\cdot 10^{-6}\frac{M}{\min}$ was obtained. Then, observed rate constants for forward and backward reactions were calculated using R_0_, $k_{f,obs}=1.22\cdot 10^{-2}\min^{-1}$, $k_{b,obs}=1.67\cdot 10^{-2}\min^{-1}$.

The absorbance at λ > 380 nm increases as time increases. This is caused scattering of light on colloidal carbon particles. They are formed continuously when pellets of activated carbon collide in the reaction vessel both with each other and with the stirring rod. The gradual increase of the background of absorption spectra slightly disturbs the absorbance readings for λ = 257 nm. This was corrected using the absorbance corresponding to the wavelength range 500–530 nm. Rh(III) bromide complex ions do not absorb light within this range of the UV-Vis spectrum.. The applied methodology is consistent with that proposed in the work of K. Kolczyk-Siedlecka et al. [45].

### 3.2. Verification of the Reaction Model

This was conducted in two steps. Firstly, the adsorption of Rh(III) bromide complex ions onto activated carbon was confirmed. Secondly, it was shown that the backward reaction is indeed desorption.

**Figure 5:**
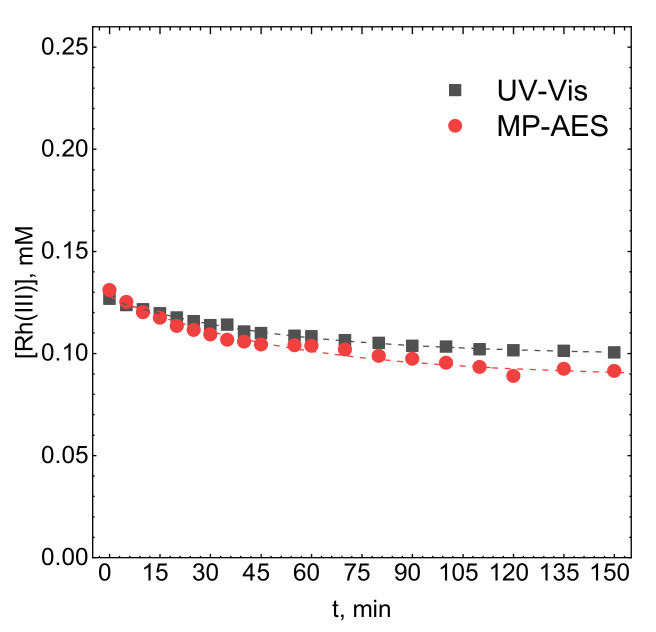
Kinetic curves obtained using the UV-Vis and MP-AES techniques. Experimental conditions: T = 50 °C, [C] = 0.5 g, pH = 1, [Br]^−^ = 0.1 M, V = 1200 rpm, [Rh(III)]_0_ ≈ 1.1 × 10^−4^ M.

Adsorption of Rh(III) bromide complex ions on activated carbon was confirmed using UV-Vis spectrophotometry. The evidence for this process was the decreasing absorbance at λ = 257 nm. This method enables rapid determination of the concentration of absorbing species and does not change the concentration of the activated carbon during the experiment. That is why it was mostly used in the described studies. Unfortunately, it is sensitive to changes in the complex structure that can be related to interaction with adsorbent. Thus, atomic emission spectroscopy, MP-AES, was employed to confirm the decreasing concentration of Rh in the studied system. The latter method determines the total concentration of Rh in the electrolyte. The kinetic curves obtained with these two techniques are compared in Figure 5.

Both kinetic curves appear to be comparable, thus, the adsorption of Rh(III) bromide complex ions on activated carbon was confirmed. The concentration difference between both methods, insignificant for the first 30 min of the experiment, then becomes apparent. This is partly because samples analyzed with MP-AES were tenfold diluted which increased uncertainty of determined concentration. More importantly, however, this result suggests that indeed some changes in the complex structure occurred. Probably, at least two forms of the complex are in equilibrium in the solution and this equilibrium is disturbed due to the adsorption. This may also suggest that adsorption and desorption rates are different for different complex forms. This will be further discussed below. Nevertheless, both kinetic curves can be approximated with the proposed model. R_0_ values are equal to 0.01914 ± 0.0023 min^−1^ and 0.01947 ± 0.0009 min^−1^ for MP-AES and UV-Vis-obtained kinetic curves respectively Because they are comparable it can be concluded that there is no significant difference between observed rate constants determined using two independent methods.

**Figure 6:**
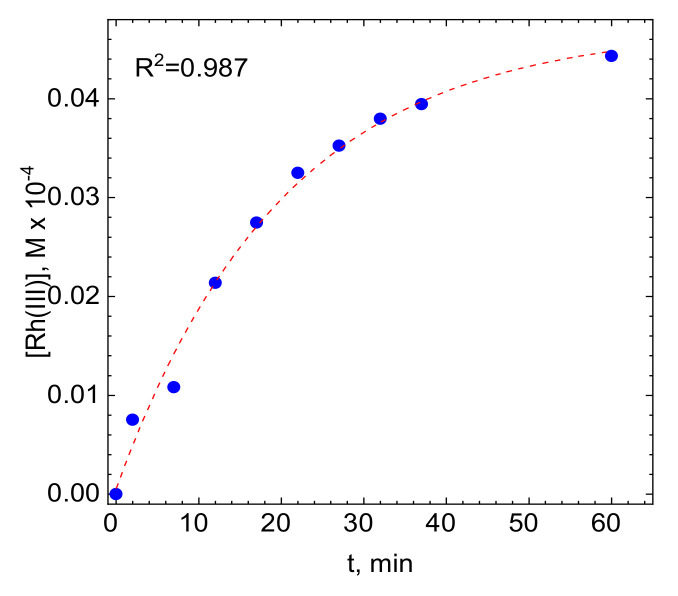
Kinetic curve obtained during the desorption test. Experimental conditions: V = 1200 rpm, [C] = 1 g, [Rh(III)]_0_ = 0 M, pH = 1, [Br]^−^ = 0.1 M T = 50 °C.

Desorption of Rh(III) bromide complex ions from the surface of activated carbon was studied spectrophotometrically in 0.1 M HBr. Increasing absorbance at λ = 257 nm means that this process indeed occurs. The kinetic curve, presented in Figure 6, approaches a certain constant value which is the equilibrium concentration of Rh(III) bromide complex ions in the solution. It was calculated that only 10% of adsorbed rhodium was subsequently desorbed. This suggests that a new equilibrium state is established in the system. On the one hand, this experiment confirms the possibility of physical, i.e., reversible, adsorption of Rh(III) bromide complex ions onto activated carbon. On the other hand, it suggests that an additional process or processes take place in the studied system.

### 3.3. Characterization of the Adsorption/Desorption Mechanisms

**Figure 7:**
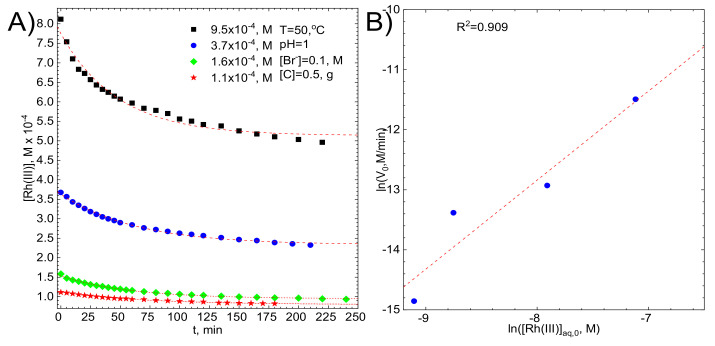
(**A**) kinetic curves for various initial concentrations of Rh(III) with fitted exponential curves, (**B**) graphical determination of the reaction order. Experimental conditions: T = 50 °C, [C] = 0.5g, pH = 1, [Br]^−^ = 0.1 M, V = 1200 rpm.

It is obvious that the initial concentration of Rh (III) bromide complex ions will influence both their equilibrium concentration in the electrolyte and the rate of the adsorption process. This is Figure 7A, where the kinetic curves obtained at T = 50 °C in stirred solution, with the stirring rate of 1200 rpm, are plotted.

The linear correlation between ln(V_0_) and ln[Rh(III)]_aq,0_, with the coefficient of determination R^2^ close to 1 was obtained Figure 7B. The slope of the fitted line, equal to 1.48 ± 0.33, confirms the initial assumption i.e., the first order of the reaction concerning the initial concentration of Rh (III) bromide complex ions.

Adsorption kinetics should also depend on the surface area of the activated carbon, provided that the system is not under diffusion control. This was verified for different concentrations of adsorbent

**Figure 8:**
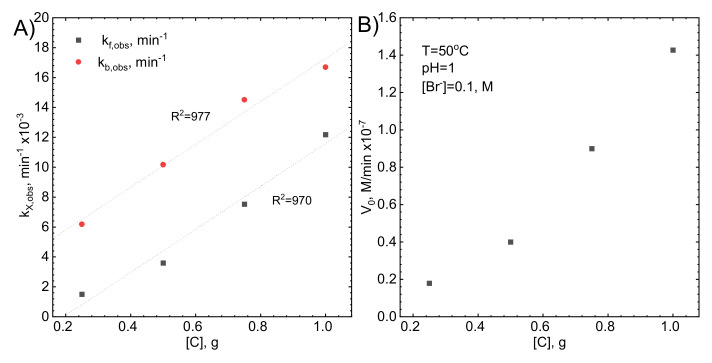
(**A**) the influence of the mass of activated carbon on the observed rate constants, (**B**) the influence of the mass of activated carbon on the initial rate, Experimental conditions: T = 50 °C, [Rh(III)]_0_~1.1 × 10^−4^ M, pH = 1, [Br]^−^ = 0.1 M, V = 1200 rpm.

Kinetic curves were approximated with Equation (11) and the observed rate constants for forward and backward reactions were determined. They both increase linearly as the amount of activated carbon in the solution increases (Figure 8A). Interestingly, there is no linear correlation between the initial rate of the process and the amount of the adsorbent (Figure 8B). This means that the adsorption mechanism is more complex than it was suggested and there is additional process. For example, Br^−^ ions can adsorb changing the stability of the Rh(III) bromide complex ions.

**Figure 9:**
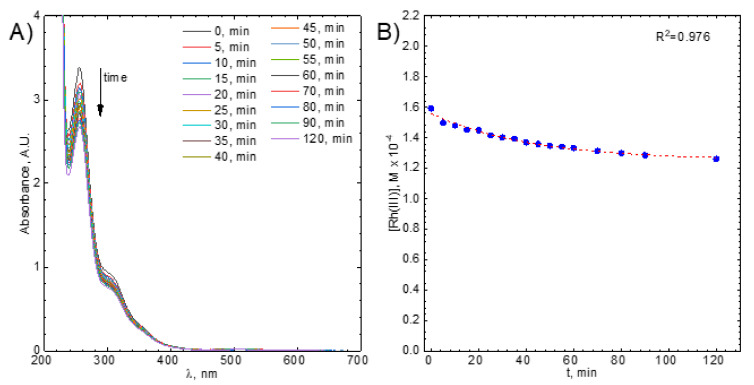
(**A**) UV-Vis spectra evolution during the adsorption process, (**B**) kinetic curve registered and fitted kinetic model. Experimental conditions: V = 1200 rpm, [C] = 0.5 g, [Rh(III)]_0_~1.1 × 10^−4^ M, pH = 1, [Br]^−^ = 0.6 M T = 50 °C.

Thus, subsequently the influence of bromide ions initial concentration on the adsorption kinetics was investigated (Figure 9A,B). For this particular experiment, their concentration was increased from 0.1 M to 0.6 M using sodium bromide.

**Figure 10:**
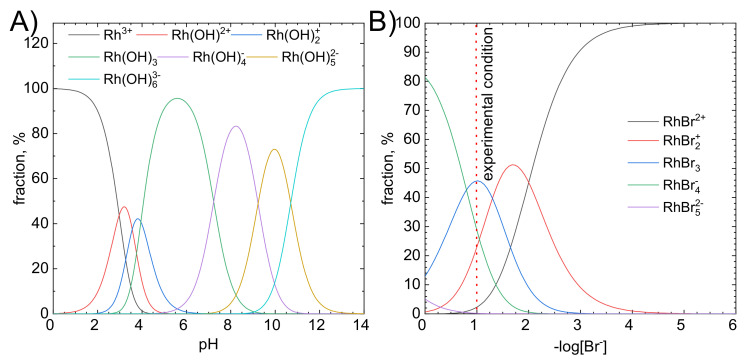
Speciation analysis of the solution, (**A**) pH impact on the complex structure and (**B**) bromide ions concentration impact on the complex structure.

First of all, the position of the maximum of the absorption band shifted from 253 to 257 nm as [Br^−^] increased from 0.1 to 0.6 M. Such a change, although small, may indicate changes in proportion between various forms of Rh(III) bromide complex ions. Thus, the speciation analysis was performed to explain obtained results (Figure 9A,B and Figure 10). The stability constants for Rh-Br system were taken from the publication of D. Cozzil and F. Pantani [34], whereas for Rh-OH system from N. Masleyi and B. Nabivanets [46]. It can be concluded that at pH = 1 hydrolysis of Rh(III) bromide complex ions virtually does not occur. Thus, hydroxide complexes of rhodium are not responsible for deviations from the proposed model. When [Br]^−^ = 0.1 M, the following forms of Rh(III) bromide complexes are in equilibrium: RhBr_2_^+^, RhBr_3_, and RhBr_4_^−^. The neutral one is dominating. When [Br^−^] increased from 0.1 M to 0.6 M, RhBr_4_^−^ became the most abundant form of the complex. At the same time, the amount of adsorbed Rh(III) ions increased by 15.8%. Therefore, at pH = 1, RhBr_4_^−^ is preferentially adsorbed onto activated carbon. This is related to the structure of activated carbon i.e., the number and kind of surface functional groups (vide infra).

**Figure 11:**
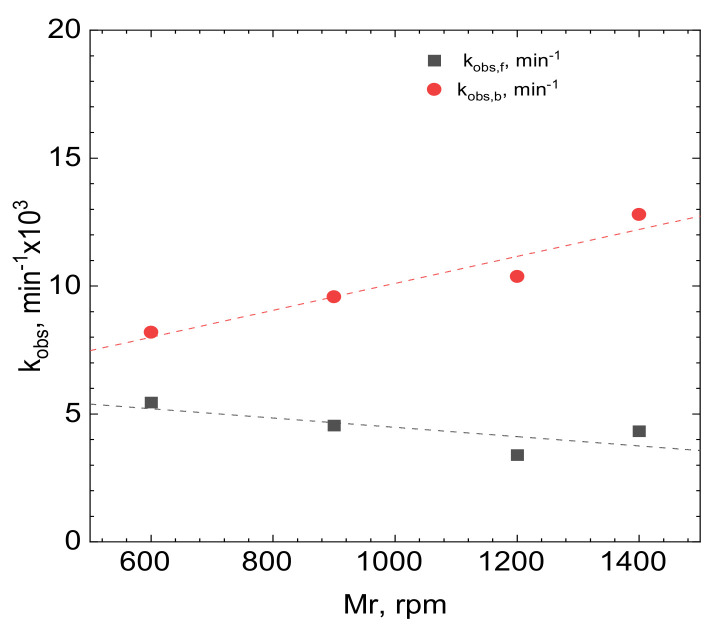
The influence of the stirring rate on the observed rate constants. Experimental conditions: T = 50 °C, [Rh(III)]_0_ ≈ 1.1 × 10^−4^M, pH = 1, [Br]^−^ = 0.1 M, [C] = 0.5 g.

The kinetics of heterogeneous processes, such as adsorption, depends on the rate of diffusion, reaction at the surface, or both of them simultaneously. The reaction model proposed in Section 3.1 neglected diffusion of the reagents because all the adsorption experiments were conducted in agitated solutions, with a stirring rate 1200 rpm. Thus, it was also necessary to identify the slowest step of the overall adsorption process. This can be done by studying the influence of the stirring rate e.g., on the observed rate Figure 11.

The results obtained indicate that the forward reaction is under activation control. Although the slope of the fitting line is slightly negative, the ANOVA test indicated that its deviation from zero is statistically insignificant, with 95% confidence level. Interestingly, the backward reaction depends on the stirring rate. This may be somehow related to oxygen dissolved in the tested solution, they were in equilibrium with air. Consequently, this might suggest that besides the adsorption, also the reduction of Rh occurs to a small extent on the carbon’s surface and reduced species when desorbed are oxidized with O_2_. This contradicts the simple model proposed. Unfortunately, the possibility of Rh reduction could not be verified experimentally. Although X-ray photoelectron spectra of activated carbon with adsorbed Rh were recorded, the signal from rhodium was comparable to the noise. That is probably because of the low concentration of rhodium in the solution. Consequently only a small amount of species adsorbed onto a high surface area of activated carbon.

Important parameters, describing the mechanism of a chemical reaction are activation energy *E_a_*, and changes of enthalpy $\Delta H^{†}$, and $\Delta S^{†}$ entropy of activation. They are usually determined graphically, using logarithmic forms of Arrhenius (Equation (14)) and Eyring-Polanyi (Equation (15)) equations, provided that the influence of temperature T on the rate constant k is known:

(14) $$\ln k=\ln A-\frac{E_{a}}{R\cdot T}$$

(15) $$T\cdot \ln\frac{k}{T}=-\frac{\Delta H^{†}}{R}+T\cdot \left(\ln\frac{k_{B}}{h}+\frac{\Delta S^{†}}{R}\right)$$

where *A* is the pre exponential factor, min^−1^, *R*, *k_B_* and h are gas, Boltzman’s and Planck’s constants. Eyring-Polanyi equation was linearized according to Lente’s suggestion because such an approach offers a better characterization of the studied system than the classical one [47].

**Figure 12:**
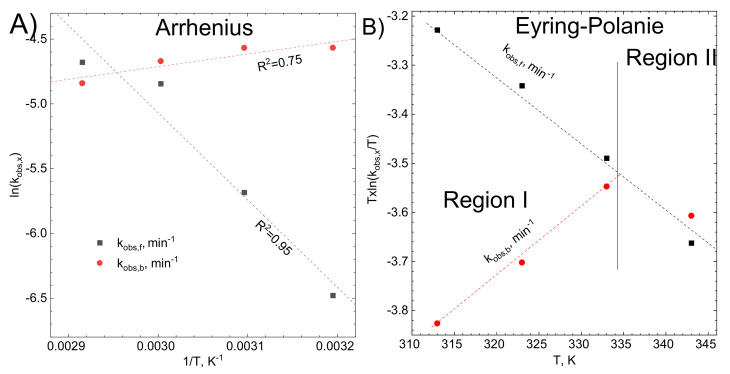
Graphical determination of (**A**) activation energy using Arrhenius model (**B**) enthalpy and entropy of activation using Eyring-Polanyi model. Experimental conditions: stirring rate 1200 rpm, [C] = 0.5 g, [Rh(III)]_0_ ≈ 1.1 × 10^−4^ M, pH = 1, [Br]^−^ = 0.1 M.

Obtained results are presented in Figure 12 and Table 1. Both forward and backward reaction fits well to the Arrhenius model, Figure 12A, and all data points were used for determination of the activation energy and the pre-exponential factor. The Eyring-Polanyi model was applicable for the adsorption reaction in the whole temperature range, but for the desorption, only the first 3 points were fitted (Figure 12B). This confirms that desorption is more complicated than it was initially assumed and consists of, at least, two processes. Both are temperature-dependent and, at T < 334 K, one of them dominates the desorption process, whereas, at T > 334 K another one. This was not observed on the Arrhenius plot meaning that the Eyring-Polanyi model is more sensitive and provides better insight into the studied system.

Uncertainties of activation energy and enthalpy were obtained directly from the fit. Uncertainty of activation entropy was calculated according to the methodology described by Lente [47]. Statistical analysis of the Eyring-Polanie equation shows that the ∆H*^†^* and *∆S^†^* uncertainties are correlated.

It can be observed that the change of enthalpy of activation is comparable to the activation energy. The change of the entropy of activation is negative. This is understandable in the case of adsorption because the number of degrees of freedom of adsorbed species decreases. Such an effect was frequently observed [37,38,48,49]. The negative change of the entropy of activation for the backward reaction is probably related to the fact, that desorbing species are not the same as the adsorbing ones. This suggests that at the surface of the activated carbon, the additional reaction takes place. The pre-exponential factor in the Arrhenius equation strongly depends on the reaction mechanism as well as the temperature [50].

It should also be underlined that the value of activation energy of the forward reaction is similar to the activation of a viscous flow of the solution [51].

### 3.4. Adsorption Isotherm

Understanding the adsorption process requires the characterization of the adsorbent. In the case of activated carbon, this means the identification of functional groups responsible for the adsorption. This can be done by comparing the XP spectra of the adsorbent prior and after adsorption. Unfortunately, in this work, this approach could not be applied probably due to the small amount of adsorbed rhodium. Identification of the adsorbing functional groups was conducted indirectly, using the XP spectrum and recently introduced model of Wojnicki-Fitzner adsorption isotherm [52]. This adsorption isotherm assumes that the adsorption of metal ions on the surface of activated carbon can be described as a chemical reaction. The number of adsorbing functional groups is directly proportional to the amount of carbon applied. Thus, it is possible to estimate their number. The Equation (1) can be given in the following form, (Equation (16)):

(16) $$m{\left[Rh(III)\right]}_{aq}+n{\left[C\right]}_{functional\;groups}\overset{k_{f}}{\underset{k_{b}}{⇌}}z[Rh{\left(III\right)}_{ads}@C_{org}]$$

The stoichiometric coefficients, m, n, and z, are introduced to this equation, contrary to Equation (1), to indicate that the adsorbing molecule can be attached to the various number of functional groups. When the equilibrium state is achieved, the rate of change of the concentration of rhodium(III) bromide complex ions in solution is zero, Equation (17)

(17) $$\frac{d{\left[Rh(III)\right]}_{t}}{dt}=0$$

Because the rates of forward and backward reactions are then equal, the following equation is obtained, Equation (18):

(18) $$k_{f}\cdot {\left[Rh(III)\right]}_{}^{\frac{m}{n}}\cdot {\left[C\right]}_{org}=k_{b}\cdot \left[Rh{\left(III\right)}_{\frac{m}{n},ads}@C_{org}\right]$$

The equilibrium constant is given in Equation (19)

(19) $$\frac{{\left[Rh{\left(III\right)}_{\frac{m}{n},ads}@C_{org}\right]}_{eq}}{{\left[C_{org}\right]}_{eq}\cdot {\left[Rh(III)\right]}_{eq}^{\frac{m}{n}}}=\frac{k_{f}}{k_{b}}=K$$

The equilibrium concentrations of Rh(III) in the solution can be determined experimentally and also calculated from the model of reaction described in the previous sections. The concentration ${\left[C_{org}\right]}_{eq}$ is unknown. Despite this, the equilibrium concentration of the reaction product can be calculated from the mass balance, Equation (20):

(20) $${\left[Rh{\left(III\right)}_{\frac{m}{n},ads}\cdot C_{org}\right]}_{eq}={\left[Rh(III)\right]}_{0}-{\left[Rh(III)\right]}_{eq}$$

Combining Equations (18) and (19) following relation can be obtained, Equation (21):

(21) $${\left[Rh(III)\right]}_{0}-{\left[Rh(III)\right]}_{eq}=K\cdot {\left[C_{org}\right]}_{eq}\cdot {\left[Rh(III)\right]}_{eq}^{\frac{m}{n}}$$

**Figure 13:**
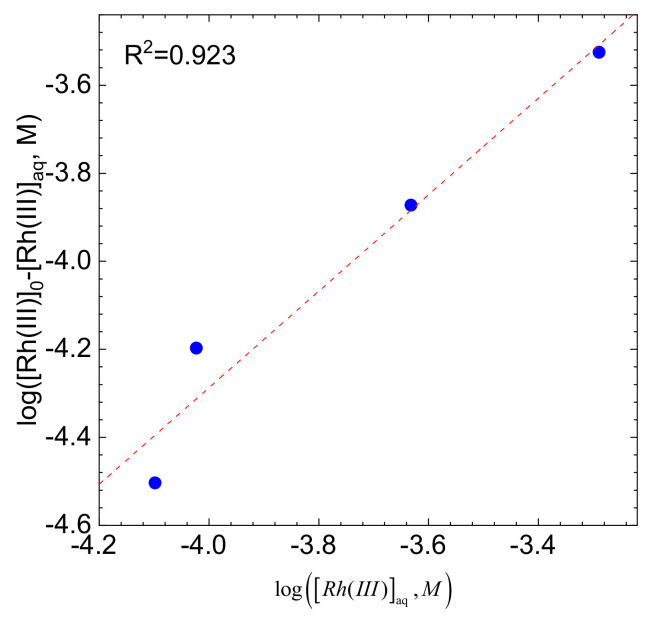
Wojnicki-Fitzner adsorption isotherm. Experimental conditions: T = 50 °C, [C] = 0.5 g, pH = 1, [Br]^−^ = 0.1 M, stirring rate 1200 rpm.

In Equation (21) three parameters i.e., *K*, ${\left[C_{org}\right]}_{eq}$ and *m/n* are unknown. It is possible to determine them graphically (Figure 13), using the logarithmic form of Equation (21), (see Equation (22)):

(22) $$\log\left({\left[Rh(III)\right]}_{0}-{\left[Rh(III)\right]}_{eq}\right)=\log(K\cdot {\left[C_{org}\right]}_{eq})+\frac{m}{n}\log\left({\left[Rh(III)\right]}_{eq}^{}\right)$$

The slope of the straight line fitting the data points is 1.1 ± 0.2 Which means that one functional group on the surface of activated carbon attaches one Rh(III) bromide complex ion. This corresponds well to the reaction order equal to 1, determined using the initial rate method. The interpretation of the intercept of the fitting line cannot be provided because its uncertainty was comparable to the fitted value.

**Figure 14:**
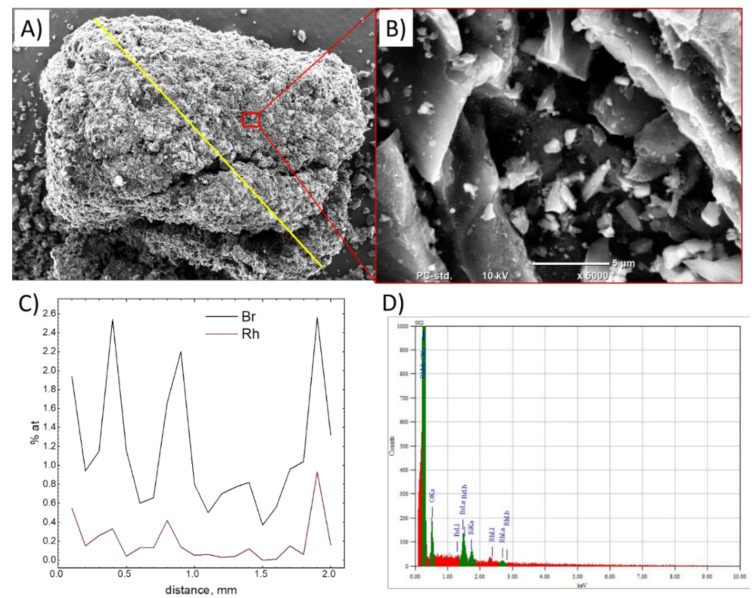
SEM image of the sample of activated carbon after adsorption test (**A**) general view indicating the line where EDS analysis was conducted, (**B**) magnification of the area where EDS point analysis was performed, (**C**) EDS line analysis results, (**D**) EDS spectrum for a selected point.

After the adsorption test, the sample of activated carbon was analyzed using SEM and XPS methods (Figure 14).

The most important result of the microscopic analysis is the distribution of Rh and Br on the sample’s surface. There is a clear correlation between rhodium and bromine content, the peak corresponding to Rh is visible in Figure 14D. The main Rh peak overlaps with the C line, therefore a less intense peak was used for the concentration estimation.

Next, the XP spectra were analysed.

**Figure 15:**
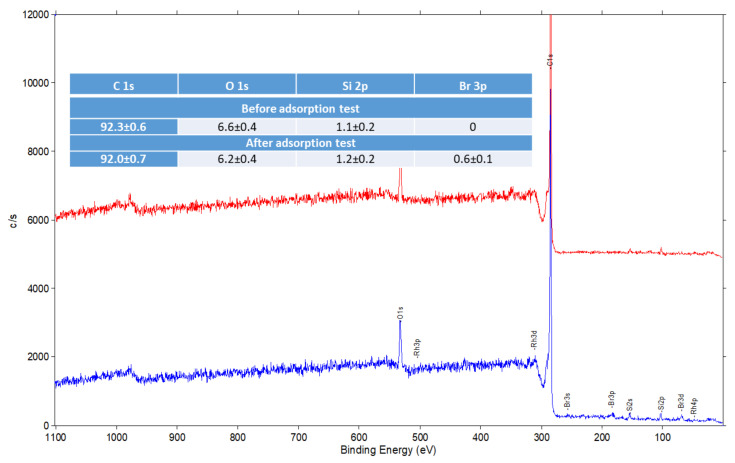
Survey XP spectra for the samples of activated carbon before and after adsorption tests.

**Figure 16:**
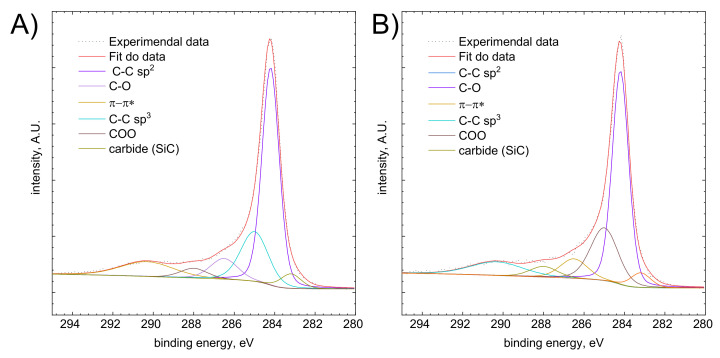
XPS spectrum of activated carbon, C1s line (**A**) before adsorption test, (**B**) after adsorption test.

In the survey spectrum of the specimenn after adsorption (Figure 15) only presence of Br was confirmed. The lack of a signal corresponding to Rh probably results from the low sensitivity of XPS method for this element. For example, the detection threshold for Pd(II) is ~10-fold lower when compared to Rh (III). The detection threshold depends on the instrument configuration i.e., the detector type. Analysis of C1s line of the XP spectrum of activated carbon is presented in Figure 16. The oxygen line was not analyzed because it is not specific i.e., there are many different compounds with similar binding energy.

The calculated fraction of determined carbon structures is shown in Supplementary Information (Table S2).

**Figure 17:**
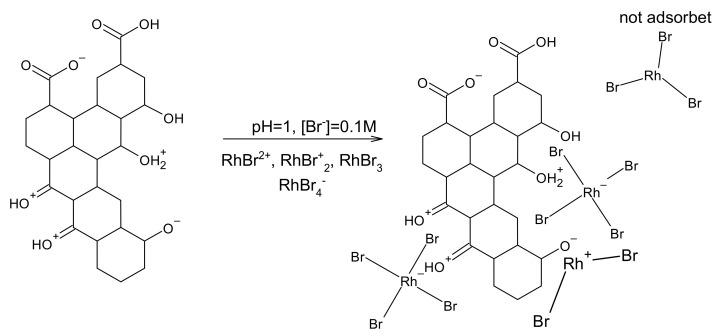
Graphical presentation of the interaction between Rh(III) bromide complexe ions with oxygen-containing functional groups.

It is usually assumed that the functional groups of activated carbon are responsible for the adsorption process. Significant amounts of C-O and COO functional groups were detected in the analysed specimen. They contain c.a. 13% of its total amount of carbon. They can be protonated or deprotonated, depending on the pH of the solution. This, in turn, affectsthe surface charge of the adsorbent [53,54,55]. This phenomenon is well known and described in the literature. It can be used for selective adsorption of selected species from the solution [27]. The presence of positively charged functional groups promotes interaction with negatively charged Rh (III) bromide complexe ions. This is shown schematically in Figure 17. It should also be noted that the content of the functional groups after adsorption experiment remained more less the same as it was before.

## 4. Conclusions

It was shown that ORGANOSORB 10—AA activated carbon absorbs Rh(III) bromide complex ions. Mechanistic studies have shown that this process is activation controlled. The positive effect of bromide ion concentration on adsorption yield was demonstrated. This suggests that negative forms of the Rh(III)-Br^−^ complexes are preferentially adsorbed. The investigated process is weakly temperature-dependent. Moreover, thanks to the Eyring equations it was possible to show that the mechanism of the process changes with temperature. It has been shown that the Wojnicki-Fitzner isotherm can be used to determine number of functional groups adsorbingRh(III) bromide complex ions. From a practical point of view, this information gives the possibility of a conscious selection of the appropriate activated carbon to obtain the highest efficiency of adsorption process. Appropriate modification of the carbon surface can also provide a high adsorption yield. This was rather low due to equilibrium condition and the small energy barrier for desorption process. A simple solution for industrial purposes is the use of a fixed-bed adsorbing column. Due to the constant change of the adsorption equilibrium, it is theoretically possible to remove most of the Rh(III) ions with a sufficiently long column.

## Figures and Tables

**Table 1:** The parameters fitted using Arrhenius and Eyring-Polanyi equations.

**Arrhenius parameters**				
**reaction**	**Intercept**	**Slope**	**E_a_ (kJ/mol)**	**A (min^−1^)**
forward	15.1 ± 3.3	−6737.8 ± 1085.9	7.0	3.61 × 10^6^
backward	−7.6 ± 0.9	976.8 ± 305.9	−1.0	5 × 10^−4^
**Eyring-Polanyi parameters**				
**reaction**	**Intercept**	**Slope**	**∆H*^†^* (kJ/mol)**	***∆S^†^* (J/mol·K)**
forward	−8200 ± 288	13.95 ± 0.89	6.81 ± 2.39	−81 ± 72
backward	866.45 ± 316.34	−13.06 ± 0.97	−7.2 ± 2.6	−306 ± 296

## Data Availability

The data presented in this study are available on request from the corresponding author.

## Supplementary Materials

Figure S1: UV-Vis spectra analysis taking into account DFT calculation, Table S1: The location of absorption maximum peaks, Table S2: Surface composition of activated carbon.
