# Supplementary material for: The Mechanism of Adsorption of Rh(III) Bromide Complex Ions on Activated Carbon
Source: Molecules. 2021 Jun 24;26(13):3862. doi: 10.3390/molecules26133862 (PMC8270305; doi:10.3390/molecules26133862)
Supplement: Supplementary file 1 [file molecules-26-03862-s001.zip › molecules-1205730-supplementary.pdf]

molecules

MDPI

Supplementary Information

The mechanism of adsorption of Rh(III) bromide complex ions on activated carbon

Marek Wojnicki[1,*], Andrzej Krawontka[1], Konrad Wojtaszek[1], Katarzyna Skibińska[1], Edit Csapó[2,3], Zbigniew Pędzich[4], Agnieszka Podborska[5] and Przemysław Kwolek[6].

[1] AGH University of Science and Technology, Faculty of Non-Ferrous Metals, Mickiewicza Ave. 30, 30-059 Krakow, Poland
[2] MTA-SZTE Biomimetic Systems Research Group, University of Szeged, H-6720 Dóm tér 8, Szeged, Hungary
[3] University of Szeged, Interdisciplinary Excellence Centre, Department of Physical Chemistry and Materials Science, H-6720, Rerrich B. tér 1, Szeged, Hungary
[4] AGH University of Science and Technology, Faculty of Materials Science and Ceramics, al. A. Mickiewicza 30, 30-059 Krakow, Poland
[5] Academic Centre for Materials and Nanotechnology, AGH University of Science and Technology, al. A. Mickiewicza 30, 30-059, Krakow, Poland
[6] Rzeszow University of Technology, Department of Materials Science, Faculty of Mechanical Engineering and Aeronautics, Rzeszow, Poland
* Correspondence: author, tel. +4812 6174126, fax: +4812 633-23-16, e-mail: marekw@agh.edu.pl (M. Wojnicki)

1. TD-DFT calculations.

Additionally, TD-DFT calculations were performed to optimize the structure of the dominant Rh (III) complex and to calculate the theoretical UV-Vis spectrum of this complex. It should be noted, that the TD-DFT calculation gives also oscillation strengths. The peaks with oscillation strengths equal to 0, were excluded. Thanks to that it was possible to reduce the number of the peaks.

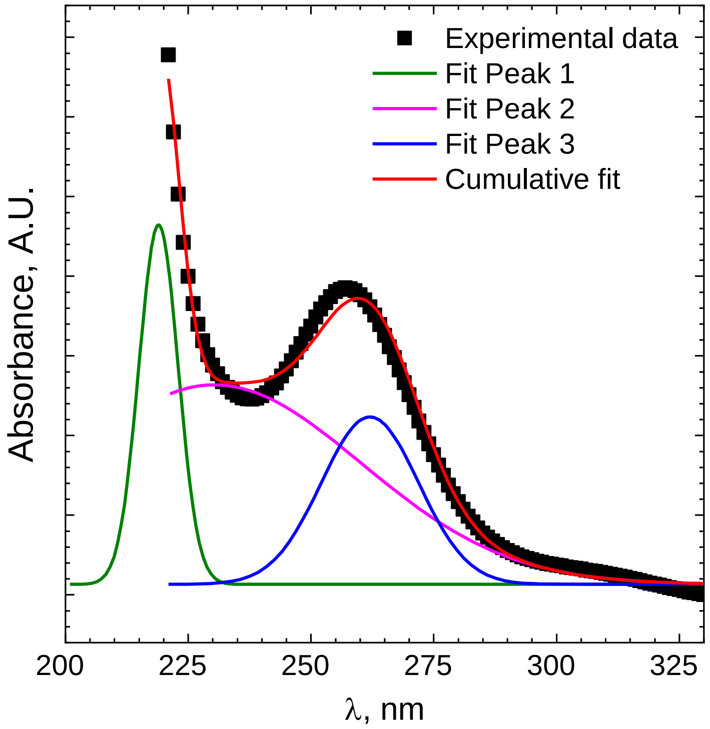

Figure S1. Comparison of experimental and calculated UV-Vis spectra of Rh(III) bromide complex ions.

It may be observed that the fit is not perfect. This is possibly related to the presence of other equilibrium forms of $RhBr_x$.

Additionally, only peaks located within the recorded spectrum were taken into account. The analyzes included a fragment of the UV-Vis spectrum with distinct peaks, i.e. the range from 220nm - 330nm. After taking into account described above assumption 4 peeks were fitted to registered UV-Vis spectra. Obtained results are shown in **Error! Reference source not found.**.

Table S1. The location of absorption maximum peaks.

Peak location calculated using DFT, nm	Peak location fitted, nm	Related species
219.91	218.94	$RhBr_3^-$
231	230.43	$RhBr_3^-$
264.63	352	$RhBr_3^-$
305.06	305	$RhBr_3^-$
305.19		

2. XPS analysis

XPS measurements were performed on two samples. Crude, i.e. the starting material, and for the carbon sample after the adsorption of Rh (III) ions. The results of C1s peak, analysis are gathered in Table S2.

Table S2. Surface composition of activated carbon.

Structure description	Binding energy, eV	Fraction, % Sample before adsorption	Fraction, % Sample after adsorption
Carbide	283.2	4.9±0.4	4.9±0.2
C-C sp2	284.2	59.7±1.3	58.6±0.9
C-C sp3	285.0	22.0±1.5	22.7±1.0
C-O	286.5	9.1±0.3	9.1±0.4
COO	288.0	4.2±0.3	4.7±0.4
π-π*	290.3	-	-

There is no statistically significant differences. This confirms that the carbon and its functional groups do not react with HBr and water. However, due to the small amount of Rh (III) on the surface, it is not visible which functional groups are involved
